# The degradation of mixed lineage kinase domain-like protein promotes neuroprotection after ischemic brain injury

**DOI:** 10.18632/oncotarget.19416

**Published:** 2017-07-18

**Authors:** Yanlong Zhou, Beiqun Zhou, Hui Tu, Yan Tang, Chen Xu, Yanbo Chen, Zhong Zhao, Zhigang Miao

**Affiliations:** ^1^ Institute of Neuroscience, Soochow University, Suzhou City, Jiangsu Province, China; ^2^ Department of Neurology, The Affiliated Hospital of Xuzhou Medical University, Xuzhou City, Jiangsu Province, China; ^3^ Department of Neurology, The Second Affiliated Hospital of Jiaxing University, Jiaxing City, Zhejiang Province, China; ^4^ Department of Anesthesia, The Second Affiliated Hospital of Soochow University, Suzhou City, Jiangsu Province, China; ^5^ Department of Neurology, The Affiliated Suzhou Hospital, Nanjing Medical University, Suzhou City, Jiangsu Province, China

**Keywords:** Necrosulfonamide, MLKL, ischemia reperfusion injury, necrosis

## Abstract

Mixed lineage kinase domain-like (MLKL) protein was recently found to play a critical role in necrotic cell death. To explore its role in neurological diseases, we measured MLKL protein expression after ischemia injury in a mouse model. We found that MLKL expression significantly increased 12 h after ischemia/reperfusion (I/R) injury with peak levels at 48 h. Inhibition of MLKL by intraperitoneal administration of NSA significantly reduced infarct volume and improved neurological deficits after 75 min of ischemia and 24 h of reperfusion. Further, we found NSA reduced MLKL levels via the ubiquitination proteasome pathway, but not by inhibiting RNA transcription. Interestingly, NSA administration increased cleaved PARP-1 levels, indicating the protective effects of MLKL inhibition is not related to apoptosis. These findings suggest MLKL is a new therapeutic target for neurological pathologies like stroke. Therefore, promoting degradation of MLKL may be a novel avenue to reduce necrotic cell death after ischemic brain injury.

## INTRODUCTION

Mixed-lineage kinase domain-like (MLKL) protein is a pseudokinase and downstream target of Receptor Interaction Protein kinase 3 (RIP3). RIP1/3 represent central players in necrosis [1–4], which is a caspase-independent programmed cell death [1, 5, 6]. Compared with apoptosis, the underlying mechanisms of necrosis remain poorly understood. Recent data show that MLKL forms complexes with RIP1 and RIP3 to induce necroptosis, a recently characterized type of programmed cell death [7]; moreover, MLKL knockdown prevents TNF-induced necrotic cell death, highlighting a critical role in the necrosis pathway [6–8].

In order to study necrotic signaling pathways, drug screens have been used to identify necrosis inhibitors like necrostatin-1 and Necrosulfonamide (NSA) [2, 7]. Necrostatin-1 is a RIP1 inhibitor that blocks TNF-induced necrosis and reduces infarct size after cerebral ischemia injury [1, 2]. NSA blocks MLKL by binding its N-terminal CC region, with similar results as MLKL knockdown by siRNA [7]. A recent study indicated that low MLKL expression is associated with poor prognosis in ovarian cancer patients [9]. This hints a role for MLKL in necrotic cell death in tumors and other diseases. Specifically, MLKL’s function in ischemic brain injury is unknown. In this study, we examined MLKL expression after cerebral ischemia reperfusion injury using a middle cerebral artery occlusion (MCAO) mouse model. Our results suggested that MLKL is a potential therapeutic target for ischemic stroke treatment.

## RESULTS

### MLKL expression showed time-dependent changes after I/R injury

**Figure 1:**
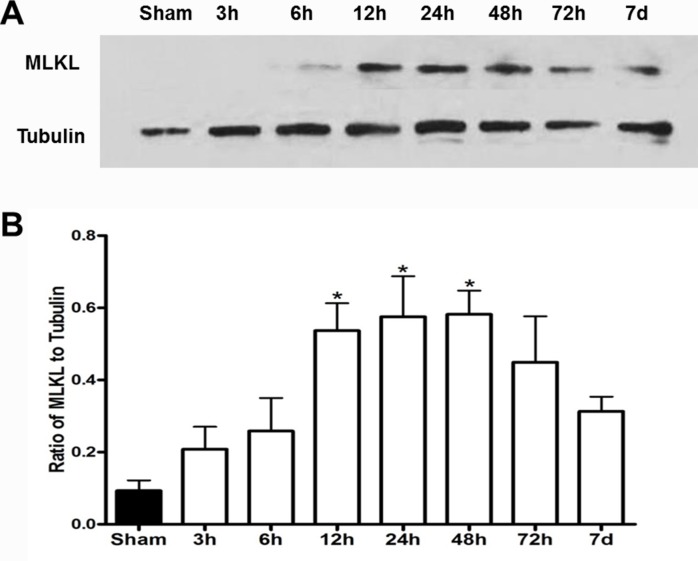
MLKL expression showed time-dependent changes after cerebral I/R injury **(A)** Representative bands of MLKL at 3, 6, 12, 24, 48, 72 h and 7 d of reperfusion after 30 min of ischemia. β-tubulin used as a loading control. **(B)** Quantitative analysis of MLKL protein was performed. *P<0.05, versus sham group, n=4.

Previous studies have indicated that MLKL plays a critical role in the process of RIP1/RIP3 complex-induced necrosis *in vitro* [7]. However, it is unknown whether MLKL expression is changed after cerebral ischemia injury. Therefore, ischemic tissues at different reperfusion time points (from 3 h to 7 d) were collected for western blot analysis. Compared with sham group, MLKL expression was significantly increased at 12 h after reperfusion, reached the peak at 48 h, and gradually decreased at 72 h and 7 d as shown in Figure 1. The results demonstrated that ischemic brain injury increased MLKL expression at the early stage of cerebral ischemia.

### MLKL expression was reduced by NSA after cerebral I/R injury

**Figure 2:**
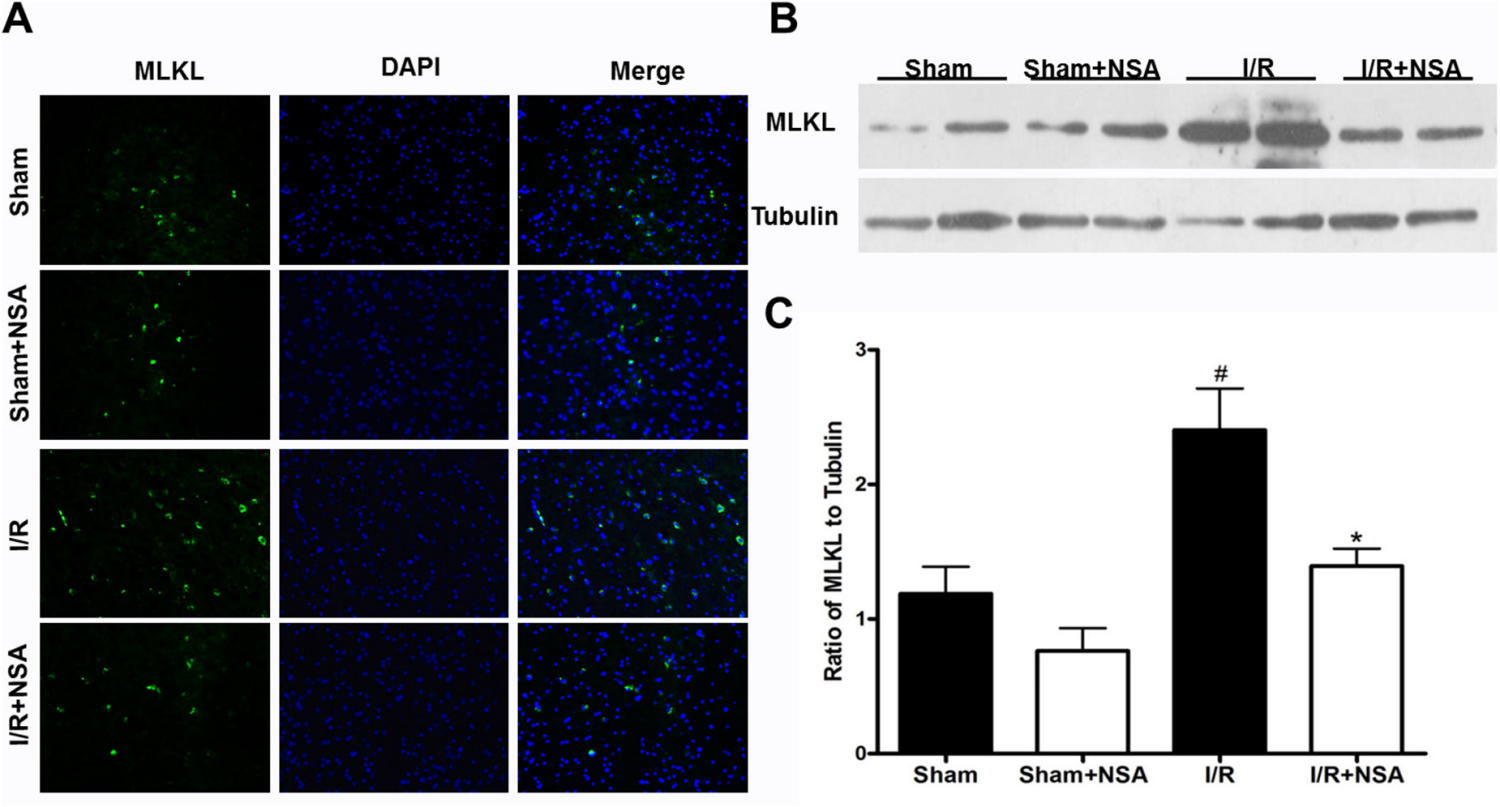
NSA treatment decreased MLKL expression after I/R injury After 30 min of ischemia and 48 h of reperfusion, ischemic brain tissues were subjected to western blot analysis and brain sections were used for immunostaining. Mice were treated with NSA (1 μmol/kg) or vehicle 30 min before ischemia. **(A)** The representative photographs show MLKL levels in ischemic brain tissues in four groups. **(B)** The expression of MLKL was determined by Western blot in four groups. **(C)** Quantitative analysis of MLKL was performed in four groups. Bars represent mean ± SEM of 4-5 brains. #, *P*<0.05 versus sham group. *, *P*<0.05 versus I/R group.

NSA is a specific MLKL inhibitor [7], it is unclear whether NSA affects MLKL expression after cerebral I/R injury. To address this question, we treated mice with NSA (i.c.v.) 30 min before MCAO. Immunofluorescent staining results showed that MLKL expression significantly increased in the I/R group 48 h after reperfusion compared with both sham group and NSA alone group (Figure 2A). In addition, MLKL significantly decreased after NSA treatment compared with I/R group (Figure 2A). Consistent with staining results, Western blot analysis showed that NSA treatment markedly reduced MLKL expression during ischemia injury (P<0.05, Figure 2B, Figure 2C).

### NSA reduced infarct volume after cerebral ischemic injury

**Figure 3:**
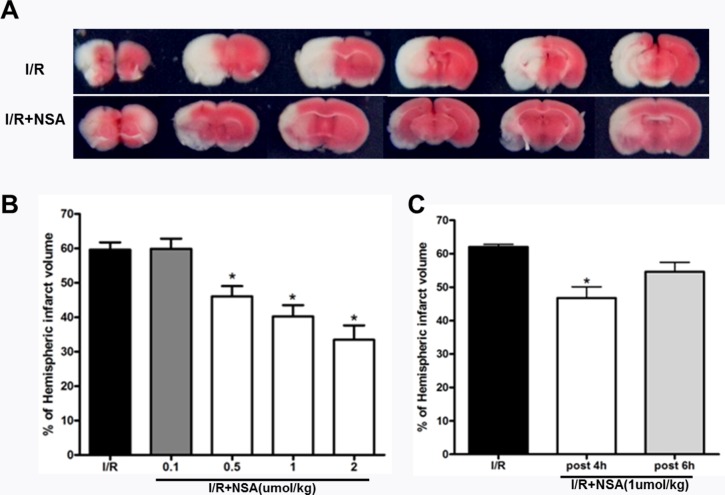
NSA treatment reduced infarct volume after cerebral I/R injury **(A)** Representative TTC-stained coronal sections in I/R group and I/R+NSA (1 μmol/kg) group. **(B)** Infarct volume with different doses of NSA pre-treatment was analyzed. **(C)** NSA post-treatment (1 μmol/kg) on infarct volume was analyzed. *P<0.05, versus I/R group. Bars represent mean ± SEM of 5-8 brains.

After neurological evaluations, brain tissue was sliced and TTC staining was performed. We found clearly large infarct volume after ischemic injury. Consistent with neurological deficit scores, NSA treatment reduced infarct volume compared with I/R group (Figure 3A). This suggested that NSA had protective effects during brain injury. To evaluate the protective efficiency, mice were treated with different doses of NSA (0.1, 0.5, 1 and 2 μmol/kg, i.c.v.). Our results showed that NSA offered protection in a dose-dependent manner (P<0.05, Figure 3B). To further examine clinical significance, we treated mice with NSA at 4 h and 6 h after reperfusion. We found NSA (1 μmol/kg) significantly reduced infarct volume at 4 h post-treatment (P<0.05, Figure 3C). However, no neuroprotection on infarct volume was seen at 6 h after reperfusion (P>0.05, Figure 3B and 3C). This suggests that NSA had a therapeutic window after ischemic injury.

### NSA improved neurological functions

**Figure 4:**
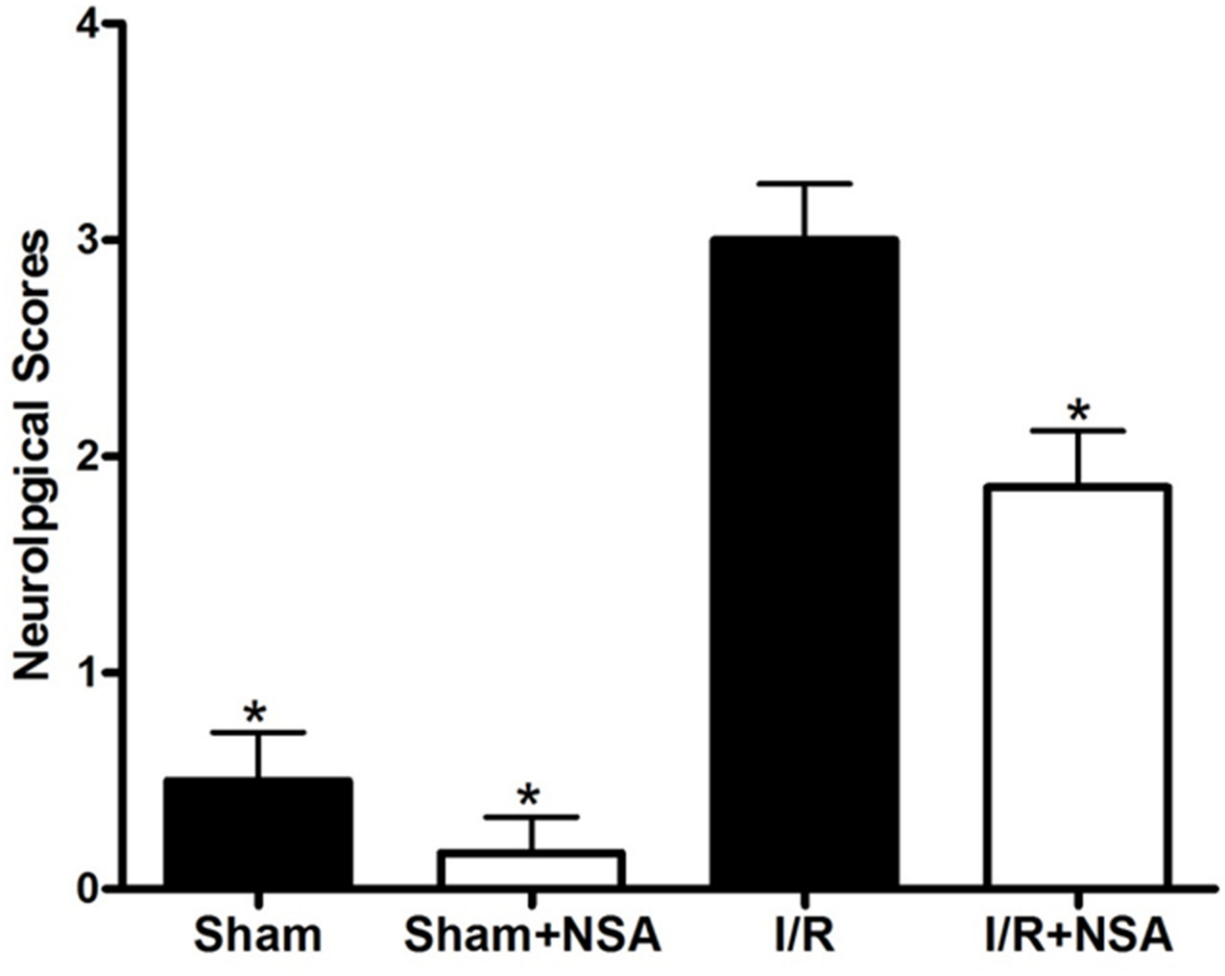
NSA treatment decreased neurological deficits after cerebral I/R injury Mice were treated with NSA (1 μmol/kg) or vehicle 30 min before ischemia. After 24 h of reperfusion and 75 min of ischemia, neurological deficit scores were evaluated in four groups. *P<0.05, versus I/R group. Bars represent mean ± SEM for samples from 5 to 8 mice in each group.

Given that MLKL expression increases after I/R injury, we speculated that MLKL inhibition will provide neuroprotection after ischemic injury. Therefore, we treated mice with NSA (2 μmol/kg, i.c.v.) 30 min before ischemia. After 24 h of reperfusion and 75 min of ischemia, neurological deficits were evaluated. As expected, cerebral ischemia injury caused significant neurological deficit scores in the I/R group. Importantly, we found that NSA treatment significantly improved neurological functions (P<0.05, Figure 4). Thus, MLKL seems to play a critical role in neuroprotection during brain injury.

### The degradation of MLKL was mediated by the ubiquitination proteasome pathway

**Figure 5:**
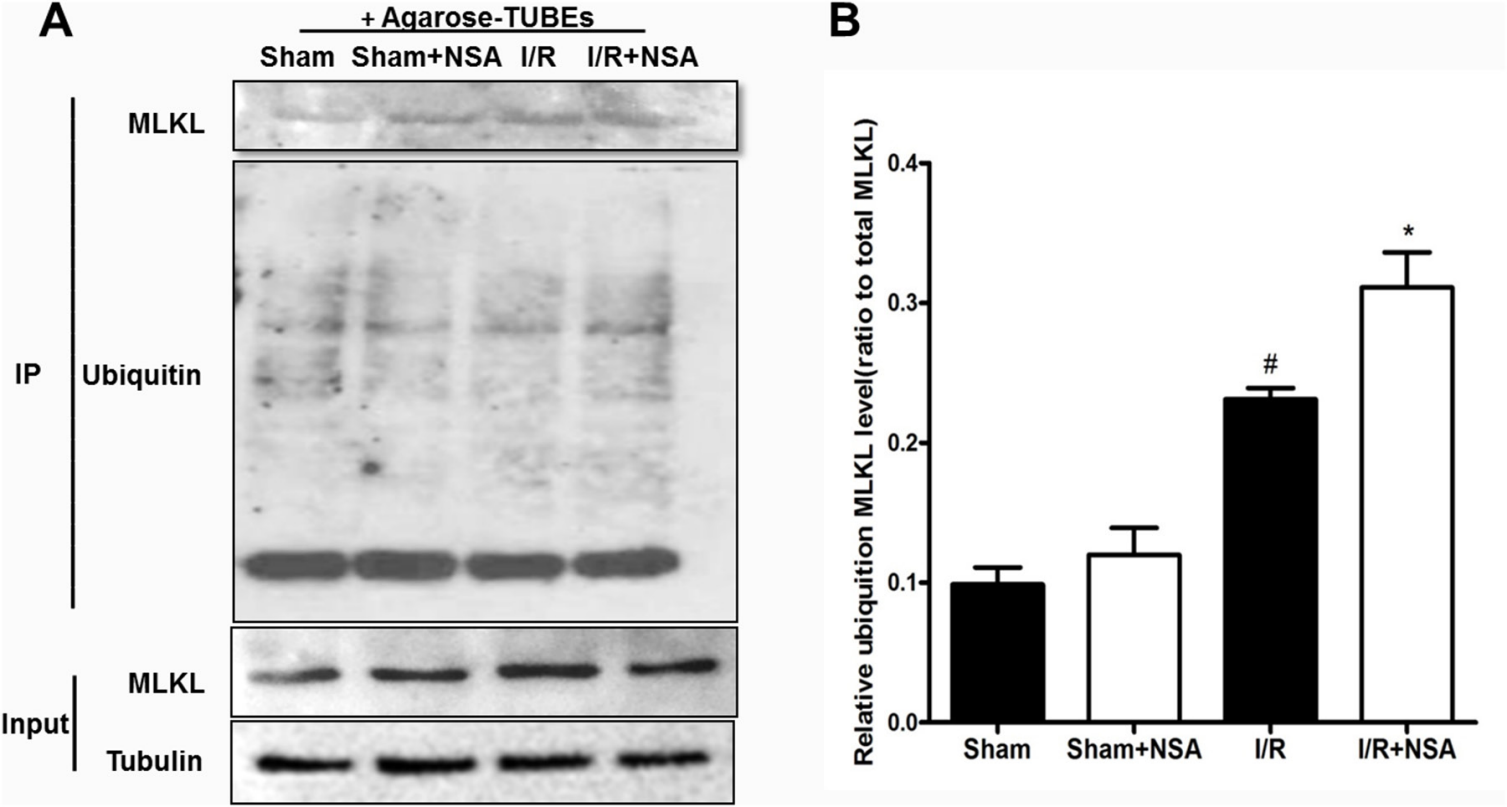
NSA treatment promoted degradation of MLKL via the ubiquitination proteasome pathway Agrose-TUBE1 was used to isolate ubiquitinated proteins from brain tissues after I/R injury. Anti-MLKL antibody detected ubiquitinated MLKL and MLKL protein input. **(A)** The representative photographs showed ubiquitinated MLKL levels in ischemic brain tissues in four groups. **(B)** Quantitative analysis of ubiquitinated MLKL was performed. Bars represent mean ± SEM of 4-5 brains. #, *P*<0.05 versus sham group. *, *P*<0.05 versus I/R group.

**Figure 6:**
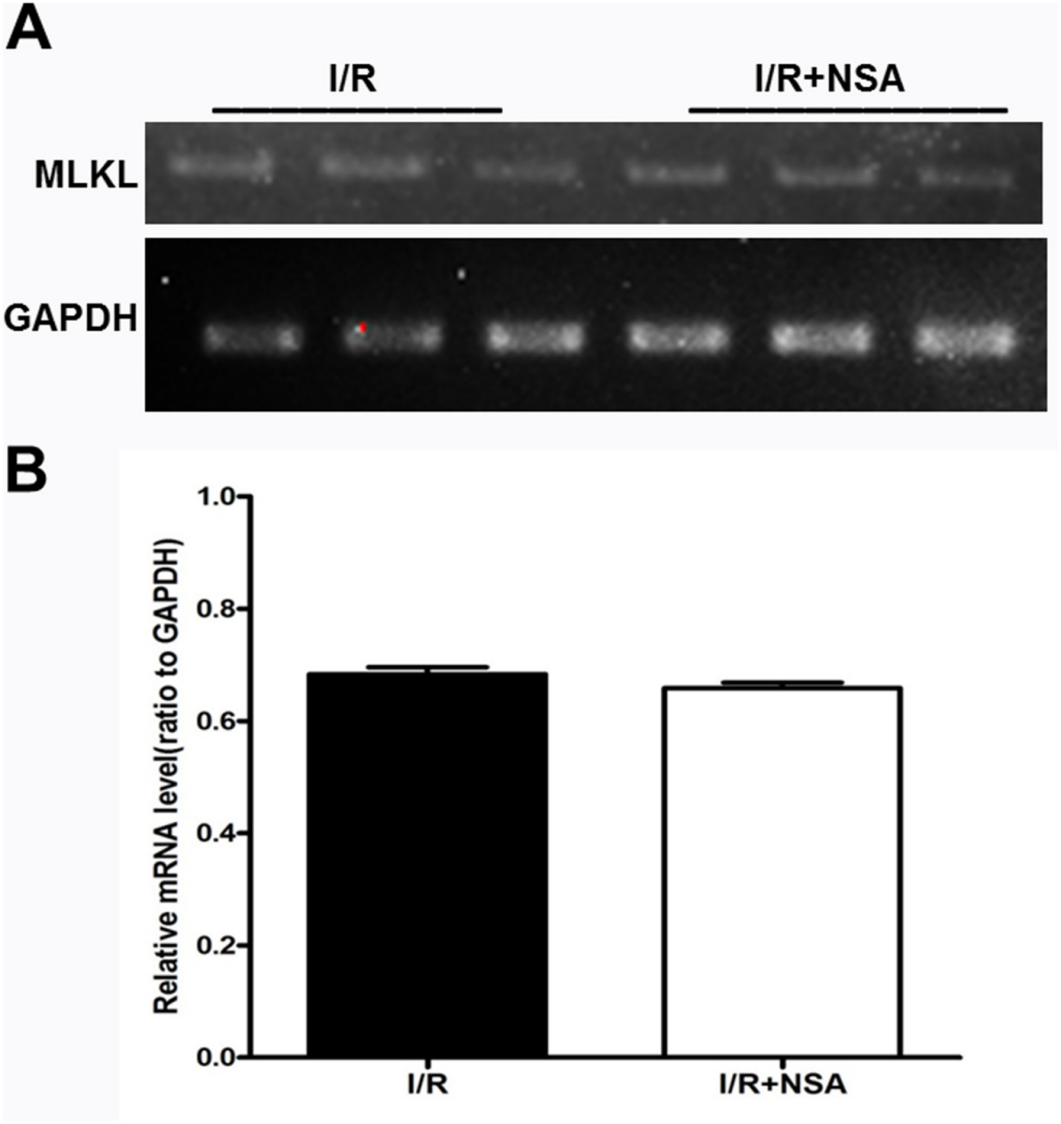
NSA did not affect the mRNA expression of MLKL after cerebral I/R injury **(A)** Representative bands of mRNA level of MLKL I/R group and I/R+NSA (1 μmol/kg) group were showed. **(B)** Quantitative analysis of mRNA level of MLKL in two groups was performed. Bars represent mean ± SEM of 4-5 brains.

A previous study showed the degradation of MLKL occurs via the ubiquitination proteasome pathway [14]. Therefore, we investigated whether NSA promotes MLKL degradation through the same process. We used tandem-ubiquitin binding entities (TUBEs) to isolate ubiquitinated proteins. As expected, our results show that I/R injury increased ubiquitinated MLKL proteins (Figure 5A and 5B). NSA treatment further increased ubiquitinated MLKL (Figure 5, P<0.05). Most importantly, MLKL mRNA did not change after NSA treatment (P>0.05, Figure 6A and 6B). This clearly demonstrates that MLKL proteins were reduced with no reduction in MLKL transcription. Thus, our data indicated that NSA promoted the degradation of MLKL protein through the ubiquitination proteasome pathway.

### Reduced MLKL associated with increased cleaved PARP-1 level after I/R injury

MLKL is considered a critical player in the necrotic cell death pathway [6, 7, 10], but it is unclear whether MLKL affects the apoptotic cell death pathway. Because Poly(ADP-ribose) polymerase-1 (PARP-1) is a substrate of caspase-3, cleaved PARP-1 is considered a marker of caspase-3-dependent apoptosis [11]. Therefore, we examined the effect of MLKL reduction on cleaved PARP-1 after I/R injury.

**Figure 7:**
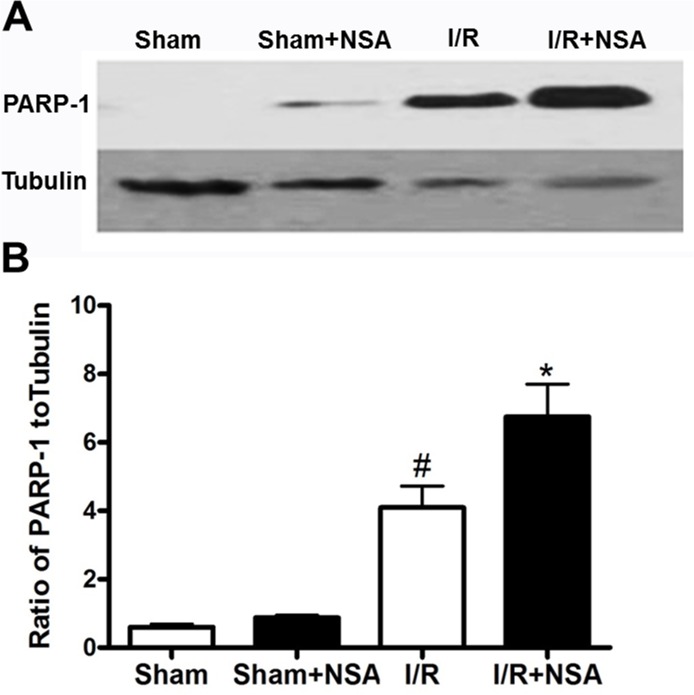
NSA treatment increased cleaved PARP-1 level after I/R injury Mice were treated with NSA (1 μmol/kg) or vehicle and the ischemic brain tissues were collected to determine cleaved PARP-1 level. **(A)** Representative bands of cleaved PARP-1 in four groups were showed. **(B)** Quantitative analysis of cleaved PARP-1 in four groups was performed. Bars represent mean ± SEM of 4-5 brains. #, *P*<0.05 versus sham group. *, *P*<0.05 versus I/R group.

We found a robust increase in cleaved PARP-1 at 24 h after I/R injury in the I/R group (P<0.05; Figure 7A). Interestingly, cleaved PARP-1 levels were even higher after NSA treatment (Figure 7A; *P*<0.05, Figure 7B). These data demonstrated the reduction of MLKL by NSA treatment blocks necrosis, but promotes apoptotic cell death.

## DISCUSSION

We demonstrate here for the first time that MLKL expression increased in ischemic brains. We also show that NSA significantly inhibits MLKL expression. In addition, reduced MLKL is associated with reduced infarct volume and improved neurological deficits after ischemic injury. Our findings suggest NSA reduced MLKL protein levels through the ubiquitination proteasome pathway and did not inhibit MLKL mRNA transcription. Interestingly, NSA also increased cleaved PARP-1, an apoptosis marker. Together, our findings suggest that MLKL protein promotes ischemia-induced cell death through a necroptotic pathway, but not an apoptotic pathway. Thus, MLKL may be an interesting therapeutic target for brain injury.

MLKL protein is a downstream target of RIP3 and is considered to be involved in ROS generation [12]. It forms a complex with RIP1 and RIP3 to induce necroptosis [7]. Kim et al suggest palmitate-induced, RIP1/RIP3/MLKL-dependent cell death might not only occur via mitochondrial ROS generation but also could be induced through other potential mechanisms such as pore forming in the plasma membrane [13]. It’s clear that RIPK3 and MLKL play critical roles in necroptosis [14, 15]. Chen et al reported that knockdown of RIPK3 or MLKL increased cell survival [16]. From these data we inferred that MLKL plays a key role in ischemic brain injury. Our experimental results support our hypothesis, showing that MLKL expression increases significantly after I/R injury. Both pre-treatment and post-treatment with NSA significantly reduced infarct volume and improved neurological deficits after ischemia. NSA interacts with human MLKL through the N-terminal region, but the region is absent in mouse MLKL [7]. Therefore, NSA should have no protection on MLKL-induced cell death in mice. However, our findings suggest that promoting degradation of MLKL is the key to the neuroprotection. We demonstrated that NSA decreased MLKL levels via the ubiquitination proteasome pathway which did not inhibit RNA transcription. The details on how NSA promotes degradation warrants further investigation.

Nec-1 blocks translocation of apoptotic inducing factor (AIF) from the mitochondria to the nucleus and suppresses the activation of PARP-1 [5, 17]. In addition, Nec-1 reverses traumatic brain injury-induced Caspase-3 activation and Bcl-2 down-regulation [18]. Nec-1 may influence different proteins involved in necrosis and apoptosis. For NSA, we speculated it would have an indirect effect on apoptosis. Due to the existence of multiple cell death pathways during ischemia, we examined the effect of NSA on cleaved PARP-1, an apoptotic marker. Surprisingly, NSA increased cleaved PARP-1, suggesting a shift from necrosis to apoptosis. Our data indicate that NSA triggered protection of necrotic cells and promoted apoptosis after I/R injury. NSA inhibits MLKL-induced necrosis and in turn drives cells to use apoptosis as an alternative cell death mechanism. The phenomenon is similar with caspase inhibition blocking apoptotic pathways that induces necrosis via TNF-α stimulus [1, 19]. This observation underscores the well-known phenomena that cells alternate between cell death systems under various environments [20].

In summary, our data demonstrate that MLKL expression was significantly increased after I/R injury. Inhibition of MLKL expression markedly reduced infarct volume and improved neurological deficits. Thus, MLKL may be a potential therapeutic target for stroke. Promoting the degradation of MLKL may represent a novel avenue for reducing necrotic cell death after ischemic brain injury.

## MATERIALS AND METHODS

### Animals

Male ICR mice weighing about 25g were purchased from SLAC Company (Shanghai, China). All mouse procedures were approved by the University Committee on Animal Care of Soochow University and carried out in strict accordance with the guidelines of Animal Use and Care of the National Institutes of Health. All surgery was performed under chloral hydrate anesthesia, and all efforts were made to minimize suffering.

### Middle cerebral artery occlusion (MCAO) model

MCAO was performed according to the method described previously [21]. Briefly, mice were anesthetized intraperitoneally (i.p.) with 4% chloral hydrate, (0.1 ml/10g body weight). We dissected the right common carotid artery (CCA), the right external carotid artery (ECA) and the internal carotid artery (ICA) through a midline neck incision. Blood flow through the CCA was then blocked via a twisted silk suture. A 6-0 nylon monofilament coated with silicon resin was introduced into the right ECA and advanced 9-11 mm. Reperfusion was achieved by withdrawing the suture after 30 min or 75 min of MCAO. Body temperature was maintained at 36.5-37.5°C by using a heating blanket throughout the procedure from the start of the surgery until mice recovered from anesthesia.

### Intracerebroventricular administration of NSA

Necrosulfonamide (NSA, Toronto, Ontario, Canada) was dissolved in dimethylsulfoxide (DMSO) for stock concentration of 10 mM. NSA was diluted to different concentrations with saline. NSA was injected via a hole (0.5 mm posterior and 1.0 mm lateral to the Bregma) by a needle with a brain stereotaxic instrument.

### 2, 3, 5-Triphenyltetrazolium chloride (TTC) staining

After neurological deficit evaluation, brains were removed quickly from the skull, cut into 6 slices and incubated with 0.2% TTC (Sigma-Aldrich, USA) at 37°C for 30 min, and fixed in 4% paraformaldehyde overnight. AlphaEase Image Analysis Software V3.1.2 (Alpha Innotech Corp., San Leandro, CA, USA) was used to analyze the infarct area in each slice. The percentage of hemispheric infarction volume was calculated as described in our previous study [22, 23].

### Neurological deficit evaluation

Neurological deficits were evaluated after 75 min of ischemia and 24 h of reperfusion based on a 5-point scale system described in our previous study [24].

### Western blot analysis

After 30 min of MCAO and 48 h of reperfusion, the whole right hemisphere tissue was collected and stored at −80°C. The 30 min time point of ischemia was chosen because minimal cell death was detected by TTC staining. Brain tissues were solubilized in lysis buffer and sonicated on ice. The tissue samples were then centrifuged and the supernatants used for Western blot analysis. The same amount of total proteins (approximately 30-40μg) were separated by sodium dodecyl sulfate polyacrylamide gel electrophoresis and then transferred to nitrocellulose membranes. After blocking with 5% dry milk in phosphate buffered saline/0.1% Tween 20 (PBST) for 2 h, blots were incubated with primary antibodies in PBST overnight at 4°C. The blots were washed with PBST and incubated with horseradish peroxidase-conjugated secondary antibody for 1 h at room temperature [25]. After washing three times, the blots were developed with ECL chemiluminescence and the immunoreactive bands captured on autoradiographic films. The densitometry of the bands was analyzed with Alpha Ease Image Analysis Software V3.1.2. Anti-MLKL goat antibody (Santa Cruz Biotechnology, USA), anti-PARP-1 rabbit antibody (ImmunoWay Biotechnology, USA), anti-ubiquitin rabbit antibody (Millipore Corporation, USA) and anti-β-tubulin mouse antibody (Sigma-Aldrich, USA) were used.

### Fluorescent immunostaining

Frozen brain sections were mounted on glass slides and blocked with buffer containing 10% goat serum, 1% bovine serum albumin (BSA), and 0.3% Triton X-100 in PBS for 1 h. The sections were then incubated with MLKL antibody in the blocking buffer overnight at 4°C. After washing with PBS for 3 times, the sections were incubated with anti-goat fluorescent secondary antibodies (Jackson ImmunoResesrch Laboratories, PA, USA) in blocking buffer for 1 h [26]. Each group contained 3 mice; every mouse had 6 slides processed; brain slices with same position from four groups were selected. All slides were examined under a fluorescence microscope (AXIO SCOPE A1, ZEISS, Germany).

### Tandem ubiquitin binding entity (TUBE) purification

Brain samples were homogenized in lysis buffer, centrifuged at 160,000g for 30 min at 4°C and then the supernatants were collected for TUBE purification. Samples were adjusted to 500 μl at a concentration of 1 μg/μl and pre-absorbed by agarose-TUBE1 (LifeSensors Inc, USA) for 12 h at 4°C with gentle shaking. Samples were spun for 5 min at 1,000×g at 4°C. Beads were further washed with lysis buffer for 3 times. The bound proteins were eluted with sample buffer containing 100 mM Tis-HCl (pH 6.8), 200 mM dithiothreitol, 4% sodium dodecyl sulfate, 0.2% bromochlorophenol blue, and 20% glycerol. The samples were heated at 96°C for 10 min and were collected for Western blot analysis.

### Total RNA extraction and RT-PCR

Brain tissues were collected and solubilized by sonication for 3 times at 5 sec each on ice in 1 ml TRIpure [27]; then 200μl of chloroform was added to the tubes. After 15 sec of the sonication and 15 min of the incubation on ice, the mixture was centrifuged at 14,000g for 15 min at 4°C. The upper solutions were collected in new tubes and mixed with equal amount isopropanol. The tubes were blended for 15 sec, then the mixture was centrifuged at 14,000g for 10 min at 4°C and isopropanol was decanted. Ice-cold 75% ethanol was added to the RNA pellet for two gentle washes. After centrifuging for 10 min, ethanol was removed and RNA pellets were dried at room temperature for 5 to 10 min. Finally, 25μl DEPC water was added to the tubes [28]. cDNA Reverse transcription was performed using the Transcriptor First Strand cDNA Synthesis Kit (Roche, Germany), and cDNA was used for PCR. GAPDH was used as an endogenous control. Primers were: MLKL (5′-GCTGCTGCTTCAGGTTTATC-3′, 5′-CGCAAGATGTTGGGATC-3′); GAPDH (5′-CATGGCCTTCCGTGTTCCTA-3′, 5′-CTTCACCACCTTCTTGATGTCATC-3′).

### Statistical analysis

All data are expressed as mean ± SEM. One-way analysis of variance followed by Tukey multiple-comparison test were used to compare differences among groups. *P*<0.05 was considered statistically significant.
